# Evaluation of the Metabolomics Profile in Charcot–Marie–Tooth (CMT) Patients: Novel Potential Biomarkers

**DOI:** 10.3390/metabo15080520

**Published:** 2025-08-01

**Authors:** Federica Murgia, Martina Cadeddu, Jessica Frau, Giancarlo Coghe, Lorefice Lorena, Alessandro Vannelli, Maria Rita Murru, Martina Spada, Antonio Noto, Luigi Atzori, Eleonora Cocco

**Affiliations:** 1 Department of Biomedical Sciences, Clinical Metabolomics Unit, University of Cagliari, 09042 Cagliari, Italy; federica.murgia@unica.it (F.M.); martina.spada@unica.it (M.S.); latzori@unica.it (L.A.); 2 Toxicology Unit, Clinical Pathology and Microbiology Laboratory, S.S. Trinità Hospital, ASL Cagliari, 09121 Cagliari, Italy; 3 School of Applied Sciences, Edinburgh Napier University, Edinburgh EH11 4BN, UK; martinacadeddu99@gmail.com; 4 Multiple Sclerosis Center, Binaghi Hospital, ASL Cagliari, 09126 Cagliari, Italy; jessicafrauneuro@gmail.com (J.F.); gccoghe@gmail.com (G.C.); lorena.lorefice@hotmail.it (L.L.); alessandro.vannelli@atssardegna.it (A.V.); mariarita.murru@aslcagliari.it (M.R.M.); ecocco@unica.it (E.C.); 5 Department of Medical Science and Public Health, University of Cagliari, 09042 Cagliari, Italy

**Keywords:** Charcot–Marie–Tooth disease, metabolomics, nuclear magnetic resonance, biomarkers, metabolic pathways

## Abstract

Background: Charcot–Marie–Tooth (CMT) is a group of inherited diseases impairing the peripheral nervous system. CMT originates from genetic variants that affect proteins fundamental for the myelination of peripheral nerves and survival. Moreover, environmental and humoral factors can impact disease development and evolution. Currently, no therapy is available. Metabolomics is an emerging field of biomedical research that enables the development of novel biomarkers for neurodegenerative diseases by targeting metabolic pathways or metabolites. This study aimed to evaluate the metabolomics profile of CMT disease by comparing patients with healthy individuals. Methods: A total of 22 CMT patients (CMT) were included in this study and were demographically matched with 26 healthy individuals (C). Serum samples were analyzed through Nuclear Magnetic Resonance spectroscopy, and multivariate and univariate statistical analyses were subsequently applied. Results: A supervised model showed a clear separation (R^2^X = 0.3; R^2^Y = 0.7; Q^2^ = 0.4; *p*-value = 0.0004) between the two classes of subjects, and nine metabolites were found to be significantly different (2-hydroxybutyrate, 3-hydroxybutyrate, 3-methyl-2-oxovalerate, choline, citrate, glutamate, isoleucine, lysine, and methyl succinate). The combined ROC curve showed an AUC of 0.94 (CI: 0.9–1). Additional altered metabolic pathways were also identified within the disease context. Conclusion: This study represents a promising starting point, demonstrating the efficacy of metabolomics in evaluating CMT patients and identifying novel potential disease biomarkers.

## 1. Introduction

Charcot–Marie–Tooth disease (CMT), also known as Hereditary Motor and Sensory Neuropathy (HMSN), is the most prevalent inherited neurodegenerative disease affecting both the motor and sensory nerves within the peripheral nervous system (PNS) [1]. Its incidence and geographical distribution vary widely across populations within regions [2]. Based on neurophysiological findings, CMT is divided into two primary forms, demyelinating (CMT1) and axonal (CMT2), although several forms with intermediate features, such as CMTX, are represented.

The clinical presentation of CMT includes musculoskeletal deformities, motor and sensory symptoms, and reduced or absent deep tendon reflexes. Generally, symptoms start in the distal region of the lower limbs and progress in a rostral direction, leading to mobility impairment [3]. Furthermore, loss of proprioception and sensory ataxia contribute to walking problems [4].

More than 100 causative genes have been associated with CMT [5]. Despite genetic heterogeneity, the pathogenic mechanism underlying Single Nucleotide Polymorphism (SNP) damage converges on the cytoskeleton of peripheral axons, resulting in axonal transport impairment [6].

The general understanding of CMT, including disease diagnosis and pathological mechanisms, has evolved significantly over the past few decades. However, currently, no therapeutic and effective drug is available for any of the CMT forms, and physiotherapy and orthotics remain the only possible therapeutic approaches [7]. Considering the clinical and genetic heterogeneity of CMT, the discovery of new biomarkers useful for early diagnosis and patient classification represents a crucial challenge to improve the correct management of affected subjects [8]. Metabolomics plays a crucial role in understanding new pathogenic mechanisms and in the discovery of new therapeutic targets or biomarkers in a disease context [9,10]. This modern approach enables the simultaneous analysis of several biomolecules (<1500 Daltons) present in different biological specimens through analytical chemistry platforms, such as Nuclear Magnetic Resonance (NMR) and Mass Spectrometry (MS) [11,12,13,14]. To date, metabolomics has been widely applied to study several polyneuropathies, particularly diabetic neuropathy. However, only a few studies have focused on Charcot–Marie–Tooth (CMT) disease [15,16,17]. The authors’ key findings were associated with significant changes in lipid metabolism, with the overexpression of key enzymes involved in lipid synthesis and triglyceride formation, signs of metabolic inflammation and sarcopenia-like features, and increased protein breakdown, which worsened with disease severity [15,16].

Despite this evidence, no widely accepted metabolite biomarkers currently exist for CMT, and our understanding of its metabolic basis remains limited. In this context, we carried out a metabolomics analysis comparing CMT patients with healthy controls. The goal was to identify key metabolite differences and characterize the metabolic profile of CMT. This could help detect molecular pathways and discover novel biomarkers and therapeutic strategies in HMSN.

## 2. Methods

This study included CMT patients (CMT) (*n* = 22) referring to the Multiple Sclerosis Centre ASL Cagliari/University of Cagliari, Sardinia, Italy, whose diagnosis was clinically and genetically confirmed. Other neurodegenerative diseases were ruled out by neurological, neurophysiological, and laboratory examinations. A control group of healthy adults (C) (*n* = 26, age > 18), demographically and ethnically matched without the presence of chronic comorbidities (such as diabetes, infectious diseases, etc.) and the use of chronic medications, was enrolled in this study.

The study was conducted in accordance with the principles of good clinical and scientific practice. Additionally, the local ethics committee approved the project (Protocol 390/10.02.2022/CE), and participants were included in the study only after they had obtained and signed the informed consent form.

### 2.1. Sample Preparation and ^1^H-NMR Acquisition

An amount of 10 mL of blood was obtained from all subjects after a night of fasting via standard intravenous sampling techniques. Samples were centrifuged at 1500× *g* for 10 min at room temperature, and the obtained serum was stored at −80 °C until the analysis. Serum samples were defrosted and centrifuged at 2500× *g* for 10 min at 4 °C, and then prepared as previously described [18,19]. Briefly, an aliquot of 400 µL of blood serum was diluted with 1200 µL of a chloroform/methanol solution (1:1, Sigma Aldrich, Gillingham, UK) and 175 µL of distilled water. Quality control (QC) samples were also used by adding and mixing an aliquot of 20 µL from each patient sample in the cohort. Samples were mixed with a vortex for 1 min and then centrifuged at 1700× *g* for 20 min at 4 °C to obtain the hydrophilic and hydrophobic phases. The hydrophilic phase was concentrated overnight.

The hydrophilic phase was resuspended in 680 µL of phosphate-buffered solution (pH = 7.3) in D_2_O (Sigma Aldrich, UK) and 20 µL of internal reference, trimethylsilylpropanoic acid (TSP) (Sigma Aldrich, UK), at a concentration of 5.07 mM. Subsequently, 650 µL of the final solution was transferred into a 5 mm NMR tube. To avoid bias during the procedure, all samples were randomized before proceeding to the instrumental analysis.

One-dimensional H-NMR spectra were collected for each sample at 300 K with a pre-saturation pulse sequence by using a Varian UNITY INOVA 500 spectrometer (11.7 T, Agilent Technologies, Inc., Santa Clara, CA, USA) equipped with a 5 mm triple resonance probe with *z*-axis pulsed field gradients and an auto-sampler in 50 locations. The spectra were recorded with a spectral width of 6000, a frequency of 2 Hz, an acquisition time of 1.5 s, a relaxation delay of 2 ms, and a 90° pulse of 9.2 µs; the total number of scans was 256. Each free induction decay (FID) was zero-filled to 64 k points and multiplied by a 0.5 Hz exponential line-broadening function [20]. The thresholds for “acceptance of shim” testing the reference peak (TSP) were <1.5 Hz.

### 2.2. Data Processing and Multivariate and Univariate Statistical Analysis

Each spectrum underwent baseline and phase correction. The data were normalized to a constant sum of 100, and each spectrum was divided into consecutive “bins” (0.04 ppm). Finally, the spectra were organized into a 48 × 146 (subjects × variables) matrix.

SIMCA-P software (ver. 16.0, Umetrics, Sweden) was employed to perform the multivariate statistical analysis. The variables were first scaled using Pareto, and the initial data analysis was conducted using the principal component analysis (PCA) method, which is fundamental for assessing sample distributions without a priori classification and detecting potential outliers (DmodX and Hotelling’s *T*2 tests were used).

Partial Least Squares Discriminant Analysis (PLS-DA) was then performed; this supervised model is designed to maximize the discrimination between samples assigned to different classes (CMT or controls). The variance and predictive ability parameters *(R^2^X, R^2^Y, Q^2^)* were evaluated to assess the suitability of the models, along with a permutation test (*n* = 400). This test performed a series of random permutations, calculating a new model with the same number of components for each one. Suppose the permutation test graph produces a new response that is considerably different from the original one; a much lower Q^2^ value is expected. Therefore, if the model does not tend to deteriorate during random permutations, it should be considered unreliable. The scores from the PLS-DA model were subjected to a Cross-Validated ANOVA test (CV-ANOVA) to determine significance (*p*-value < 0.05). To study a possible linear relationship between the metabolic profile (matrix X, predictor variables, e.g., metabolites) and the clinical parameters (matrix Y, dependent variable, such as age), PLS projection to latent structure regression models was carried out [21].

The most significant variables were then extracted from the volcano plot for each model and identified and quantified using Chenomx NMR Suite 7.1 (Chenomx Inc., Edmonton, Alberta, Canada) [22]. Metabolites were utilized to perform the univariate statistical analysis (GraphPad Prism software version 9.3.1, GraphPad Software, Inc., CA, USA). The Mann–Whitney *U*-test was performed and ROC curves were built to confirm the sensitivity and specificity of the molecules. Moreover, Effect Size Cohen’s d was also calculated for the discriminant metabolites.

### 2.3. Metabolic Pathway Analysis

Metabolic pathways were analyzed via the web server MetaboAnalyst 5.0 (www.metaboanalyst.ca), generating an exhaustive metabolomic data analysis, visualization, and interpretation. This methodology enabled the identification of a correlation between significant metabolite changes and their corresponding metabolic network [23].

## 3. Results

A total of 48 subjects were recruited: 22 people that were affected by CMT syndrome (CMT), and 26 healthy controls (C). Out of the 22 CMT patients, 4 were diagnosed with CMT1, 11 with CMT2, 2 with CMTX, and for 5 patients, no diagnostic information was available (Table 1).

In 6 patients (4 CMT1 and 2 CMT2), the molecular diagnosis was not yet identified. For the CMTX group, the genetic variants were CX32Val63Phe, Cx32 Arg142Trp, and Cx32 Val139Met. In the CMT2 group, 4 patients carried the P0 Ser44Phe genetic variant, and 3 patients the pHSP27 R127W genetic variant. All the remaining CMT1 patients carried the PMP22 duplication.

A total of 44 hydrophilic molecules were identified in the NMR spectra. Four CMT samples contained an unknown contaminating molecule and were eliminated from this study. By using the bins matrix, the PCA model was evaluated using the CMT group and controls to identify potential outliers within each group that could affect further data analysis. Subsequently, PCA and PLS correlation models were performed to identify any statistically significant differences or correlations between gender and age among the samples.

**Figure 1:**
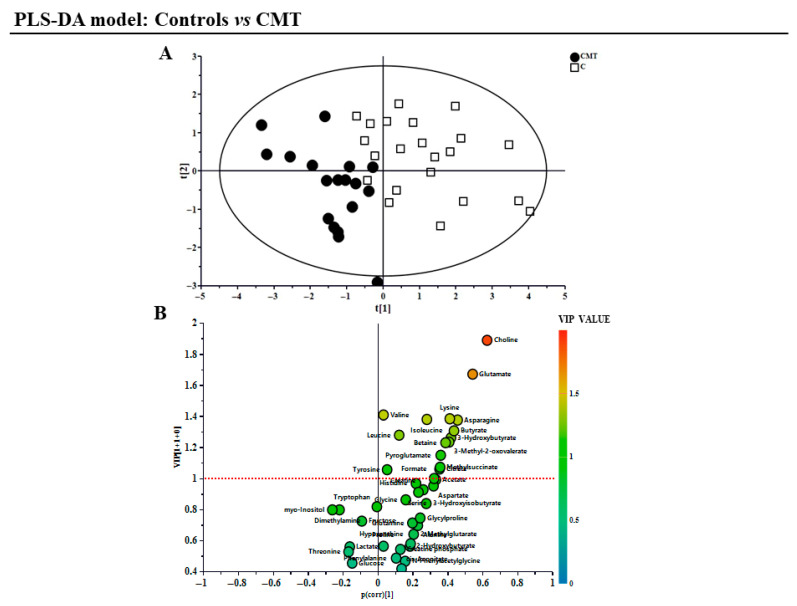
Supervised PLS-DA and a relative volcano plot showing the different sample groups, generated using Simca software. (**A**) Supervised PLS-DA showing CMT (black dots) and controls (white squares), where R2X = 0.3, R2Y = 0.7, and Q2 = 0.4; *p*-value = 0.0004. The data represent the distribution of the different classes of samples among the graph, which, as can be seen, are not evenly distributed. Instead, they are separated, with CMT samples on the left and control samples on the right. (**B**) A volcano plot of metabolites and their corresponding VIP value. Metabolites above the red dotted line (VIP value ≥ 1) were tested for concentration and statistical significance between the CMT and control groups. The data display the volcano plot obtained by plotting all 41 metabolites considered, along with their corresponding VIP values. Through this analysis, it was possible to verify which metabolites distinguish and discriminate between the control group and the group with patients affected by CMT disease.

The PLS-DA model was used to further detect a clearer distribution of controls and CMT samples within the graph (Figure 1, R^2^X = 0.3; R^2^Y = 0.7; Q^2^ = 0.4; *p*-value = 0.0004). The permutation test was completed to evaluate the randomness and the presence of over-fitting data within the model under investigation (intercepts R^2^ = 0.3, Q^2^ = −0.25). Through the volcano plot, metabolites showing a VIP value ≥ 1 were selected and analyzed using the non-parametric Mann–Whitney *U*-test, which was performed to determine the statistical significance between the two groups.

**Figure 2:**
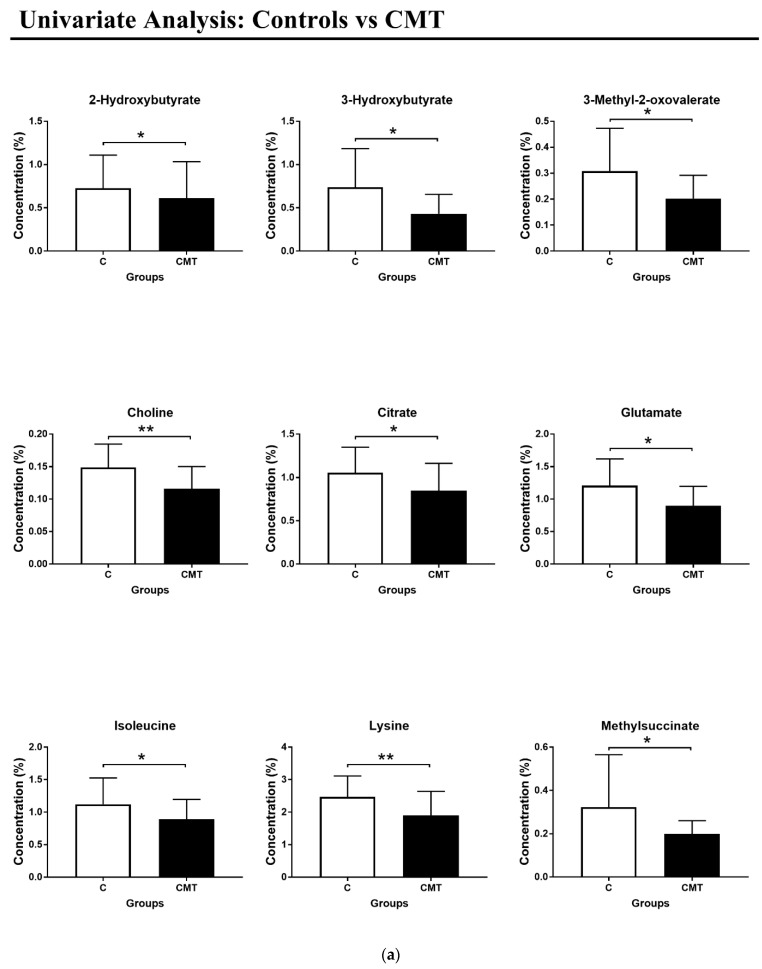

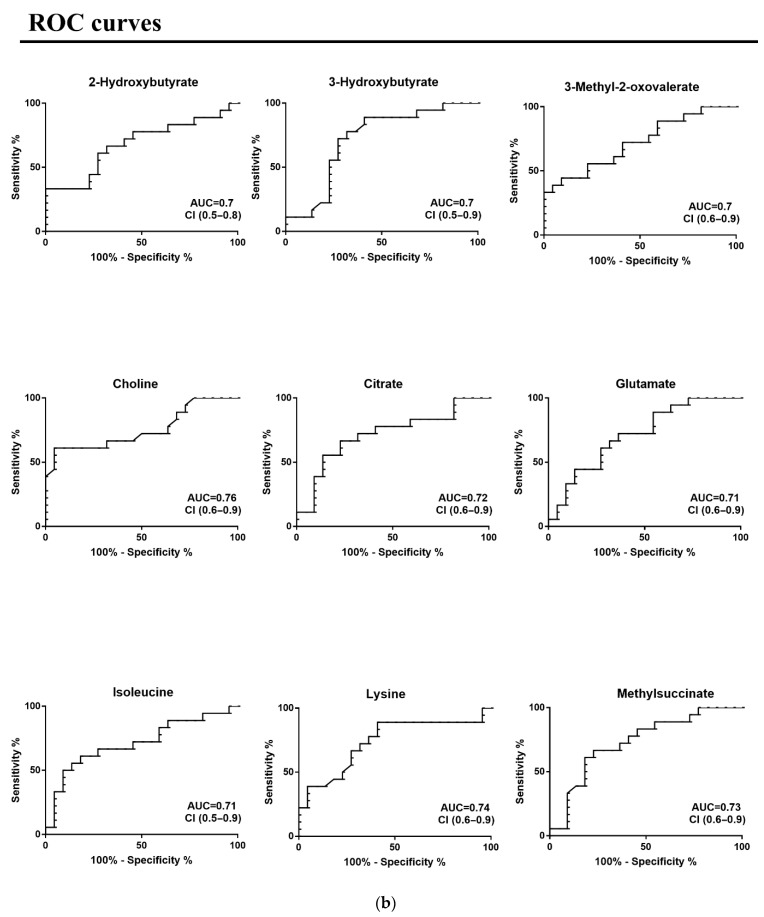
(**a**) Box plots of concentrations (%) of significant metabolites between the CMT and control groups. The data represent the difference in metabolite concentrations (%) between the control (white) and CMT groups (black). Among all metabolites with a VIP value greater than 1, nine of them showed a statistically significant difference between controls and CMT patients. A Mann–Whitney U-test was performed to evaluate statistical significance. The * symbol indicates a statistically significant difference between the two groups; more specifically, * corresponds to *p*-value < 0.05, whereas ** corresponds to *p*-value < 0.005. (**b**) ROC curves of significant metabolites.

By using the discriminant metabolites, Receiver Operating Characteristic (ROC) curves were built to evaluate the sensitivity and specificity of the model. Results are presented in Figure 2a,b and Table 2. Discriminant metabolites were 2-hydroxybutyrate, 3-hydroxybutyrate, 3-methyl-2-oxovalerate, choline, citrate, glutamate, isoleucine, lysine, and methyl succinate.

**Figure 3:**
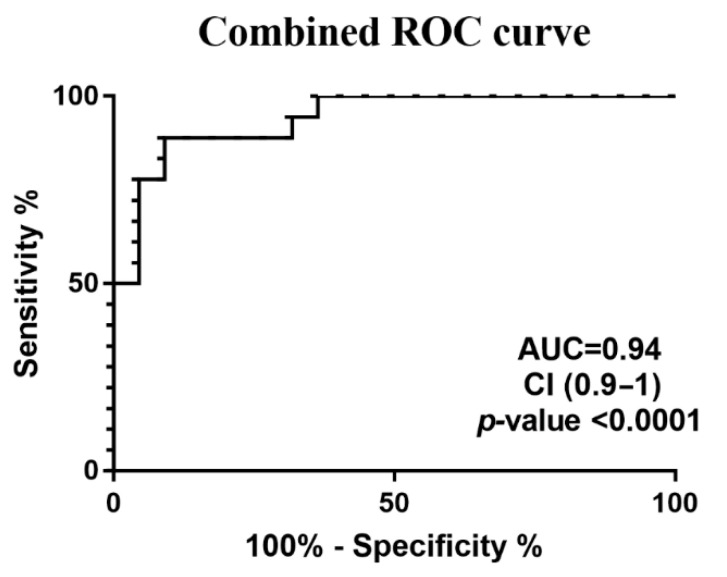
Combined ROC curve obtained by adding the significant metabolites. The data represents the ROC curve obtained by combining individual concentrations from significant metabolites.

To test whether these nine metabolites when combined could better discriminate and metabolically describe the CMT group from the controls, their concentrations were merged to build a single ROC curve achieving AUC = 0.9 (Figure 3).

A further supervised PLS-DA model was tested to observe potential metabolic differences between CMT subtypes (CMT1, CMT2, and CMTX were not considered, because only two patients were affected by this form). The sample’s distribution within the graph was visually distinct (CMT1 *n* = 4; CMT2 *n* = 10), but not statistically significant.

**Figure 4:**
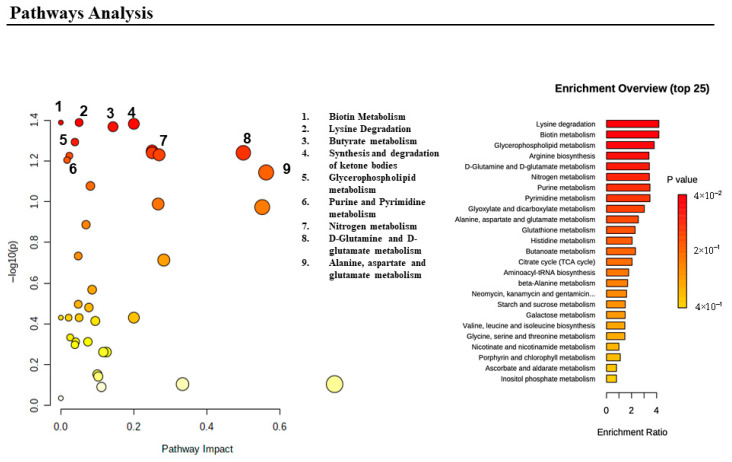
Schematic representation of the pathway analysis, generated via MetaboAnalyst web server. Data represents the top metabolic pathways being altered among the CMT sample group.

The presence of altered metabolic pathways was analyzed via MetaboAnalyst 5.0, which enabled extensive metabolomic data analysis, visualization, and interpretation. The most altered metabolic pathways were related to amino acid metabolism such as lysine degradation, as well as glutamine, glutamate, alanine, and aspartate metabolism. Additionally, pathways involved in energetic homeostasis including the synthesis and degradation of ketone bodies were also affected. Moreover, other alterations were related to biotin metabolism, purine and pyrimidine metabolism, butyrate metabolism, nitrogen metabolism, and glycerophospholipid metabolism. The correlation between significant metabolite changes and their corresponding metabolic network is displayed in Figure 4.

## 4. Discussion

This study represents one of the first comprehensive metabolomic investigations of CMT disease, aiming to identify potential disease biomarkers through a comparative analysis of discriminant metabolites between patients with CMT and healthy controls. Despite CMT being the most common inherited neuropathy worldwide, the field still lacks reliable molecular markers capable of characterizing the presence and progression of the disease [16].

The integration of metabolomics into the study of CMT holds the potential to uncover common biochemical pathways disrupted in various forms of the disease. Beyond biomarker discovery, it offers a valuable tool for elucidating pathogenic mechanisms, enhancing diagnostic precision, and informing individualized treatment strategies.

Our metabolomics analysis revealed significant alterations in the levels of nine key metabolites—2-hydroxybutyrate, 3-hydroxybutyrate, 3-methyl-2-oxovalerate, choline, citrate, glutamate, isoleucine, lysine, and methyl succinate. Combined, these metabolites yielded strong discriminative power between patients and controls (AUC = 0.94, *p* < 0.0001). Further pathway analysis revealed these compounds as functionally interlinked, suggesting systemic metabolic reprogramming in CMT.

One of the most notable findings was the decrease in ketone bodies (3-hydroxybutyrate) and citrate, both of which play a central role in energy metabolism. Ketone bodies serve as critical energy substrates for neural tissues under carbohydrate-limiting conditions, and their reduced levels in CMT patients may reflect impaired lipid utilization or mitochondrial dysfunction [24]. On the other hand, in the brain, ketone bodies play a neuroprotective role as key signaling agents, drivers of protein post-translational modification, and modulators of inflammation and oxidative stress, thereby preserving neuronal synaptic function and structural stability [25,26,27,28]. Mitochondrial dysfunction is increasingly recognized as a standard feature in neurodegenerative and neuroinflammatory disorders, including CMT, where deficits in energy production contribute to axonal degeneration and progressive disability [29,30].

Citrate, a pivotal intermediate in the tricarboxylic acid (TCA) cycle, is essential for ATP synthesis. Its depletion implies mitochondrial inefficiency, potentially leading to neuroinflammation and neuronal death. Given the high energy demands of neurons, disruptions in the TCA cycle severely compromise axonal integrity, contributing to the chronic progression of CMT [31].

A notable feature of our results was the generalized reduction in amino acid levels. Glutamate, isoleucine, and lysine, each integral to protein synthesis and cellular metabolism, were significantly decreased. Glutamate, in particular, is essential for Schwann cell function and myelination. Its reduced levels suggest a dysregulation of glutamine synthetase (GS), an enzyme converting glutamate to glutamine. Previous studies have shown GS overexpression in disease states, which may contribute to improper Schwann cell phenotyping and demyelination, a hallmark of CMT1 subtypes [32,33].

Reduced isoleucine levels may indicate diminished activity of 2-methyl-3-hydroxybutyrate-CoA dehydrogenase, a key enzyme in branched-chain amino acids (BCAAs) and lipids. Deficiencies in this enzyme have been implicated in progressive neurological decline, supporting a role for metabolic insufficiency in CMT pathogenesis [34,35]. Similarly, lysine deficiency may reflect altered lysine acetylation, an important regulatory mechanism in mitochondrial biogenesis and neuronal survival. Impairments in this process are associated with mitochondrial dysfunction and axonal degeneration.

Choline, another metabolite found reduced in our cohort, plays diverse roles in membrane phospholipid synthesis, acetylcholine production, and epigenetic regulation via DNA and histone methylation [36,37]. Decreased choline availability may affect neuronal membrane integrity and neurotransmission, particularly in axonal CMT forms, where presynaptic dysfunction has been observed.

Together, these findings underscore a broad disruption of energy production, amino acid metabolism, and membrane maintenance in CMT patients. The metabolite profile not only reflects neurodegenerative processes but also reveals mechanistic insights into mitochondrial stress, altered myelination, and impaired neurotransmission.

To date, few studies in the literature have addressed CMT disease from the metabolomics point of view: Soldevila et al. aimed to identify novel biomarkers in both plasma and skin biopsies that reflect disease severity in CMT1A, using metabolomics analysis (plasma) and proteomic profiling (skin). They enrolled CMT1A patients (stratified as mild, moderate, or severe) and healthy controls and applied MS analysis to test 194 metabolites in plasma. Twelve key metabolites effectively classified disease severity; metabolite trends were linked with elevated markers of protein catabolism (e.g., dipeptides, tryptophan, urobilinogen) and sphingolipid-linked inflammatory signals (e.g., sphingosine-1-phosphate, lysophosphatidylcholine) and decreased leucine, important for muscle biogenesis, consistent with sarcopenia-like presentation in CMT1A. Skin proteome changes support an early mitochondrial and oxidative stress deficit, reinforcing the metabolic and neurodegenerative interplay in CMT1A.

Another recent study by Setlere et al. aimed to analyze plasma metabolite concentrations in a CMT cohort and compare them to healthy controls. They detected 33 metabolites with the MS approach, from which acetyl carnitine was found elevated and glycine and valine were found decreased in CMT patients compared to controls. However, further analysis revealed poor disease predictive abilities of the detected metabolites for any CMT group.

Considering our metabolomics study together with those present in the literature performed on plasma samples, the emerging features in CMT disease seem to be characterized by inflammation, muscle catabolism, lipid signaling alterations, and compromised bioenergetic capacity.

## 5. Conclusions

The results obtained highlight the ability of the metabolomics approach to effectively discriminate between CMT patients and controls, as well as the potential to predict the association of subjects with the CMT or control class based solely on their blood serum profile. Several metabolites with strong potential as diagnostic or prognostic biomarkers have been found. However, several limitations are present in this study. Further validation in larger, genetically stratified cohorts will be essential to confirm their clinical utility. Nevertheless, this approach allows us to generalize these results to all the HSNMs, which clearly show common pathogenetic pathways.

Diet, lifestyle, or medications could play a role as confounding factors in metabolomic data. On one hand, in an exploratory study, it might be desirable to carefully select patients to obtain a homogeneous cohort; however, especially in a rare disease, this can be difficult. Interestingly, clinical and lifestyle information can be integrated into the database and used as Y variables to correlate the metabolic profile and investigate possible influences.

Another limitation of the investigation is the use of NMR spectroscopy alone, without combining it with mass spectroscopy. Despite NMR spectroscopy being a very accurate and efficient tool for identifying metabolites, its significant disadvantage consists in its lower sensitivity compared to MS. Perhaps a follow-up study involving both techniques could yield more accurate and comprehensive results in terms of metabolites being detected, which could ultimately lead to a more complete analysis and evaluation of the metabolomics profile of CMT disease.

Our exploratory study represents the first step of a complex path, but despite several limitations, we achieved a good discriminating capacity with the method, as nine novel potential biomarkers of the disease were discovered. This suggests enormous potential from both diagnostic and therapeutic perspectives. The use of one or more discriminating metabolites could be helpful not only for identifying pre-pathological or disease stages but also lays the foundations for further investigations that could verify the possibility of detecting differences between the various forms of the disease and to evaluate whether therapy can modify the metabolic status of the treated subjects and how it can be used for monitoring the response to therapy.

## Figures and Tables

**Table 1:** Demographic and clinical features of individuals used in this study. CMT patients are sub-divided by diagnosis (CMT1, CMT2, and CMTX).

Demographic Characteristics			
	CMT (*n* = 22)	C (*n* = 26)	*p*-Value
Male/Female	8/14	14/12	>0.05 * (ns)
Mean age (years) ± SD	49.3 ± 11.6	52.4 ± 9.6	>0.05 ** (ns)
Age range (years)	32–75	22–73	
CMT diagnosis	CMT1 = 4 CMT2 = 11 CMTX = 2		

* Pearson’s Chi-squared test: X-squared = 0.84738, df = 1, *p*-value = 0.3573. ** Wilcoxon signed-rank test: V = 98.5, *p*-value = 0.05196.

**Table 2:** Metabolites discriminating against the CMT group, being statistically decreased in comparison to the control group. AUC = Area under the Curve; CI = Confidence Interval.

Metabolites	CMT	Effect Size Cohen’s D *	*p*-Value	ROC Curve			
				AUC	Standard Error	95% CI	*p*-Value
2-Hydroxybutyrate	-	0.30	0.05	0.68	0.09	0.5–0.8	0.05
3-Hydroxybutyrate	-	0.86	0.02	0.72	0.08	0.5–0.9	0.02
3-Methyl-2-oxovalerate	-	0.84	0.02	0.72	0.08	0.6–0.9	0.02
Choline	-	1.33	0.005	0.76	0.08	0.6–0.9	0.01
Citrate	-	0.69	0.02	0.72	0.08	0.5–0.9	0.02
Glutamate	-	0.87	0.02	0.71	0.08	0.5–0.9	0.02
Isoleucine	-	0.64	0.02	0.71	0.09	0.5–0.9	0.02
Lysine	-	0.93	0.01	0.74	0.08	0.6–0.9	0.01
Methylsuccinate	-	0.66	0.01	0.73	0.08	0.6–0.9	0.01

* Effect sizes: very small (0.1 ≤ d < 0.2), small (0.2 ≤ d < 0.5), medium (0.5 ≤ d < 0.8), large (0.8 ≤ d < 1.2), very large (d > 1.2).

## Data Availability

Data available on request.
